# Relationship of the Macular Ganglion Cell and Inner Plexiform Layers in Healthy and Glaucoma Eyes

**DOI:** 10.1167/tvst.8.5.27

**Published:** 2019-10-17

**Authors:** Sasan Moghimi, Nima Fatehi, Andrew H. Nguyen, Pablo Romero, Joseph Caprioli, Kouros Nouri-Mahdavi

**Affiliations:** 1 Glaucoma Division, Stein Eye Institute, David Geffen School of Medicine, University of California at Los Angeles, Los Angeles, CA, USA; 2 Hamilton Glaucoma Center, Shiley Eye Institute, University of California San Diego, San Diego, CA, USA

**Keywords:** glaucoma, ganglion cell layer, inner-plexiform layer, SD-OCT

## Abstract

**Purpose:**

To explore factors influencing the inner plexiform layer (IPL) in healthy subjects and to test the hypothesis that IPL thickness is preferentially decreased in glaucoma as compared with ganglion cell layer (GCL) thickness.

**Methods:**

Ninety-nine glaucomatous eyes and 66 healthy eyes (165 subjects) underwent macular spectral-domain optical coherence tomography (SD-OCT) imaging and GCL and IPL were segmented creating 8 × 8 arrays of 3° × 3° superpixels. The central 24 superpixels were categorized into three levels of eccentricity (∼1.5°, 4.5°, and 7.5° from the foveal center). Linear mixed models were used to determine predictive parameters for IPL thickness in healthy subjects and to explore the influence of diagnosis of glaucoma on IPL thickness taking into account the effect of GCL thickness and other covariates.

**Results:**

Being located at 4.5° eccentricity predicted thicker IPL compared with 1.5° eccentricity (*P* < 0.001) in multivariable models in healthy subjects, whereas older age (*P* = 0.001) and Asian ethnicity (*P* = 0.021) were associated with thinner IPL. Diagnosis of glaucoma was not associated with thinner IPL regardless of eccentricity after accounting for age and ethnicity. The results were similar when only eyes with mean deviation greater than –6 dB were analyzed.

**Conclusions:**

Ethnicity and distance from the fovea are the main determinants of IPL thickness in the central macula. Preferential thinning of the macular IPL, compared with GCL, could not be detected in this study regardless of glaucoma stage.

**Translational Relevance:**

There is no evidence for preferential thinning of the macular IPL in glaucoma compared with GCL based on currently available SD-OCT–imaging technology.

## Introduction

Early detection of glaucoma and its progression is important to treat the disease in a timely manner and to prevent visual disability. Glaucoma is caused by retinal ganglion cell (RGC) axonal injury at the level of the optic nerve head. However, there is growing evidence that measurement of the RGC mass in the macula can contribute to our understanding of neural loss in glaucoma as more than 30% of the RGCs are located within 16° from the fovea.^1^ Macular thickness parameters have been demonstrated to perform well for detection of early glaucoma.^2^,^3^ Moreover, variability of various macular structural parameters is very low and comparable to that of RNFL parameters.^4^,^5^ Improvements in the resolution of spectral-domain optical coherence tomography (SD-OCT) images and development of segmentation algorithms have made measurement of individual retinal layers, including the inner plexiform layer (IPL) and ganglion cell layer (GCL) possible.^6^–^8^ Miraftabi et al.^4^ recently reported consistently low and uniform variability for thickness measurements across the central macular region derived from SD-OCT imaging in 102 glaucomatous patients and 21 healthy subjects. Recent work by Hood et al.^9^ and other investigators has demonstrated that significant loss of central macular RGCs can occur before the appearance of clinically detectable visual field loss. Animal studies have shown that dendritic shrinkage may occur before RGC death.^10^,^11^ Tan and colleagues^8^ found that both GCL and IPL decreased in glaucoma eyes compared with a healthy control group. Our recent work on structure–function relationships in a group of healthy eyes and eyes with a wide range of glaucomatous damage showed slightly stronger structure–function relationships for GCL compared with IPL.^2^ Two recent studies also showed that IPL thickness was able to discriminate glaucoma eyes from healthy subjects with reasonable accuracy.^12^,^13^

De Moura et al.^14^ reported that in healthy eyes, the IPL became progressively thicker as compared with GCL with increasing eccentricity. They also found that, in advanced glaucoma, the residual thickness of the IPL was larger than the residual thickness of the GCL (i.e., IPL had a higher measurement floor). This is consistent with the report by Miraftabi et al.^4^ that confirmed a smaller dynamic range and higher measurement floor for IPL. Although the absolute thickness of the inner retinal layers (GCL and IPL) changes with eccentricity in the healthy retina, the IPL to GCL relationship may also change with glaucomatous damage. A relatively lower IPL compared with GCL thickness in glaucoma eyes would be suggestive of early dendritic shrinkage.

The goals of the current study were (1) to explore factors influencing the IPL thickness in healthy subjects, and (2) to test the hypothesis that IPL thickness may be preferentially decreased in glaucoma compared with GCL thickness.

## Methods

Participants in ongoing studies at The Glaucoma Advanced Imaging Laboratory at Stein Eye Institute were included. Ninety-nine eyes of 99 glaucoma patients and 66 eyes of 66 healthy subjects were included. Among glaucoma patients, 89 eyes (89 patients) had perimetric glaucoma and 10 eyes (of 10 patients) had preperimetric glaucoma. All procedures were carried out according to the tenets of the Declaration of Helsinki and met Health Insurance Portability and Accountability Act regulations requirements. Written informed consent was obtained from all the participants after explanation of the procedures to be used. The studies were approved by the institutional review board at the University of California Los Angeles.

All participants had measurement of best-corrected visual acuity, slit-lamp biomicroscopy, refraction, corneal pachymetry, Goldmann applanation tonometry, gonioscopy, dilated fundus examination, biometry (IOLMaster; Carl Zeiss Meditec, San Leandro, CA), and achromatic visual field testing (SITA standard 24-2 fields with the Humphrey Field Analyzer; Carl Zeiss Meditec, Inc., Dublin, CA), and macular SD-OCT imaging (Spectralis OCT; Heidelberg Engineering, Heidelberg, Germany). Glaucoma was diagnosed by the attending physician based on the presence of glaucomatous optic nerve damage (i.e., presence of broad rim thinning or notching, cup-to-disc asymmetry >0.2) and was confirmed by reviewing optic disc photographs by a glaucoma specialist (KNM). A visual field defect was considered to be present on 24-2 fields if both of the following criteria were met: (1) glaucoma hemifield test outside normal limits, and (2) four abnormal points with *P* < 5% on the pattern deviation plot, both confirmed at least once.^15^ Preperimetric glaucoma was defined as presence of glaucomatous optic nerve damage in the absence of an established visual field defect as indicated above. Patients with astigmatism more than 3 diopters (D) or significant retinal or neurologic disease were excluded. Diabetic patients were included in the original study only if there were no signs of retinopathy. Healthy participants had a normal eye exam, including open angles, normal appearing optic discs, and 24-2 standard achromatic visual fields.

### Imaging Protocol

**Figure 1:**
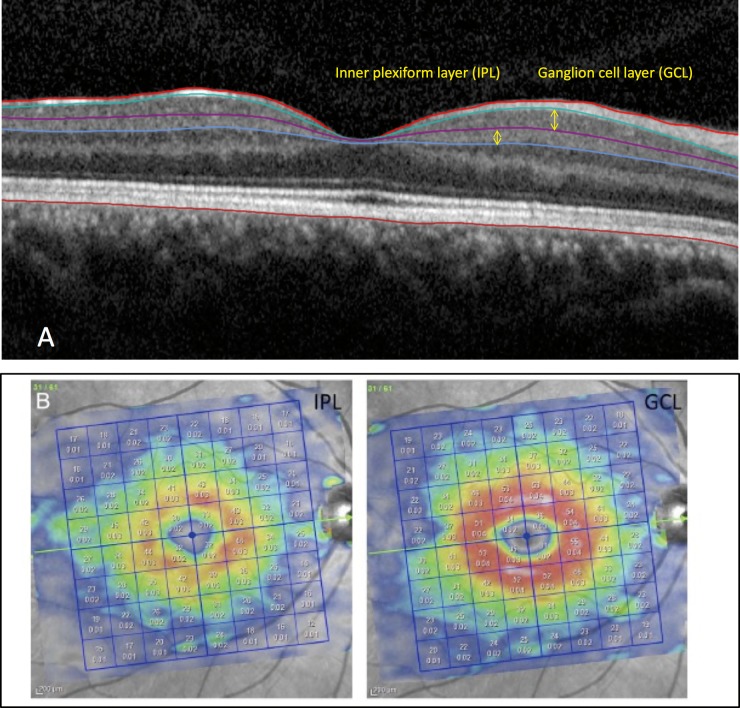
(A) An OCT B-scan after automated segmentation demonstrating the GCL and IPL. (B) Examples of macular images demonstrating the IPL and GCL thickness measurements displayed as 8 × 8 arrays of 3° × 3° superpixels after segmentation, derived from the Posterior Pole algorithm of the Spectralis SD-OCT device (Heidelberg Engineering) in a healthy eye. The top numbers within the superpixels provide the superpixel thickness and the bottom numbers represent the superpixel volume.

**Figure 2:**
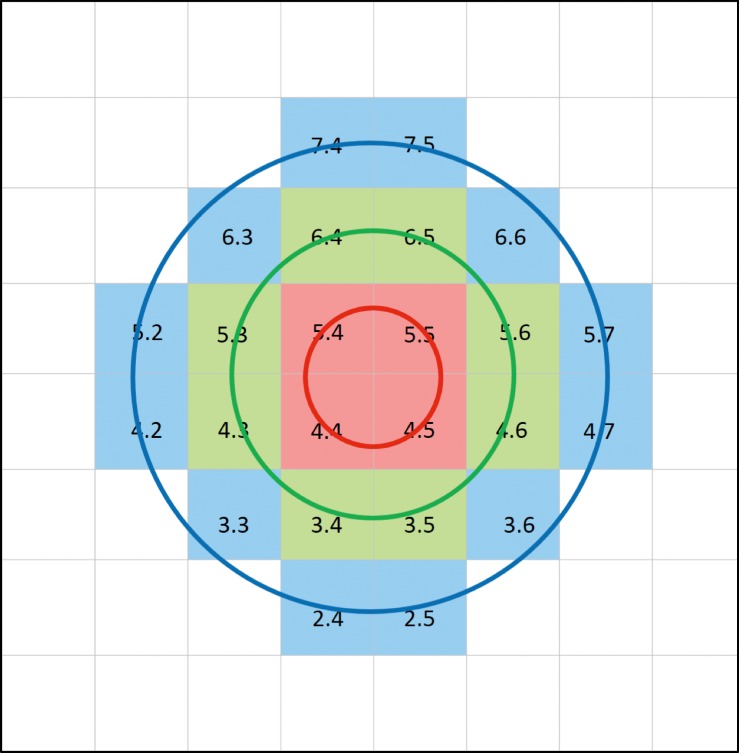
Central 24 superpixels from the Posterior Pole algorithm of the Spectralis SD-OCT were categorized into three eccentricities according to distance from the fovea. Red superpixels: approximately 1.5° from the foveal center (red circle); green superpixels: approximately 4.5° from the foveal center (green circle); blue superpixels: approximately 7.5° from the foveal center (blue circle).

The Posterior Pole algorithm of the Spectralis SD-OCT was used to obtain 30° × 25° volume scans of the macula (61 B-scans spaced ∼120 μm) centered on the fovea. The central 24° × 24° of the measurement cube is segmented by the software and data are presented in an 8 × 8 array of 3° × 3° superpixels. Each B-scan was repeated between nine and 11 times to improve image quality (Fig. 1). Segmentation of individual retinal layers was performed with the Glaucoma Module Premium Edition software (Heidelberg Engineering, Heidelberg, Germany), and the data were exported as extensible markup language files. Images were reviewed for segmentation errors and image artifacts by one of the investigators. Any obvious segmentation errors were manually corrected using the SD-OCT device's built-in software. If more than two B-scans within the central 24° of any individual volume scan were of inadequate quality or showed poor segmentation, that eye was excluded from analyses. A low-quality B-scan image was defined as quality factor less than 15 dB, the presence of more than 10% missing data or inadequate segmentation or any artifacts, such as mirror artifacts. Also, images were excluded if there was any evidence of inner retinal disease, including epiretinal membrane, subclinical cystoid macular edema, and so on. Presence of mild or scattered drusen with no apparent effect on the inner retina was not a criterion for exclusion. The macular layers of interest in this study were the IPL and GCL. The central 24 superpixels of the macular image were selected for this study as this area represents the thickest region of the GCL. The central 24 superpixels were divided into three eccentricities (circles) according to distance from the foveal center, located approximately 1.5°, 4.5°, and 7.5° from the foveal center for circles 1 through 3, respectively (Fig. 2).^2^ When both eyes of a subject were eligible, only the right eye was included.

### Statistical Analysis

Histograms and contingency tables were used for exploring the distribution of the numeric and categoric variables, respectively. Scatter plots of IPL versus GCL were constructed for the 3 eccentricities with the GCL at individual superpixels as the predictor for the IPL thickness.

To determine factors influencing the relationship of IPL to GCL, we first carried out univariable and multivariable predictive models in healthy subjects with various demographic and biometric factors entered into the model. Parameters with a *P* value < 0.15 in univariable models were included in the multivariable models. Linear mixed models were then applied to all patients to investigate the relationship of the IPL to GCL thickness adjusting for the covariates found in the predictive models of IPL thickness in healthy subjects. The mixed models accounted for the clustering of the 24 superpixels within an individual eye. Given the significant influence of eccentricity on the relationship of the IPL with GCL thickness, all regression analyses were performed separately for each eccentricity. The main finding of interest in these models was the influence of a diagnosis of glaucoma on IPL thickness after accounting for all other covariates, including the GCL thickness. These models included the interaction between diagnosis and GCL thickness.

Because IPL thickness measurements reach the measurement floor earlier compared with GCL in glaucoma patients, all regression analyses were carried out after excluding superpixels with GCL thickness less than 30 μm so that a linear model could still be fit to the data. To test the hypothesis of linearity of IPL on GCL above GCL thickness of 30 μm, we compared linear models, including splined GCL thickness as the predictor (instead of GCL thickness), with the linear models, including nontransformed GCL thickness as a continuous variable in the model. Likelihood ratio (LR) test was then used to compare these pairs of models at each eccentricity. We also plotted the IPL to GCL ratio as a function of 24-2 visual field mean deviation (MD) and applied a Lowess fit. If early IPL thinning existed in early glaucoma eyes, the Lowess fit for glaucoma eyes would be located lower than that for healthy eyes. A *P* value > 0.05 on the LR test provides strong evidence that the assumption of linearity is valid. We also estimated and compared areas under the receiver operating characteristic curves (AUCs) for global and superior/inferior hemiretinal GCL and IPL thickness measurements. All statistical analyses were performed with the software Stata (version 14.0; StataCorp, College Station, TX).

## Results

No eye was excluded because of poor image quality and all B-scans had a quality factor greater than 15 dB. The demographic and clinical characteristics of the study sample are presented in Table 1. The mean (±SD) age was 65.4 (±10.0) years in the glaucoma group compared with 52.6 (±13.0) years in the healthy subjects (*P* = 0.001). Glaucoma eyes had an average visual field MD of −7.1 (±5.2) dB compared with −0.2 (±1.2) dB in healthy eyes. Also, glaucoma eyes had longer axial length and mildly thinner central corneal thickness on average (*P* = 0.001 and *P* = 0.029, respectively). Visual field MD was better than −6.0 dB in 52 eyes (early glaucoma, 52.5%), was between −6.0 and −12.0 dB in 29 eyes (moderate glaucoma, 29.3%), and worse than −12.0 dB in 18 eyes (advanced glaucoma, 18.2%).

**Table 1:** Demographic and Clinical Characteristics of the Glaucoma and Control Groups

Number (Patients, Eyes)	Glaucoma (99 Eyes Of 99 Patients)	Healthy (66 Eyes Of 66 Subjects)	*P* Value
Sex, female:male	59:40	39:27	0.515
Age, y, mean ± SD	65.4 ± 10.0	52.6 ± 13.0	0.001
Spherical equivalent, D, mean ± SD	0.2 ± 10.7	−0.9 ± 2.1	0.446
Race, n (%)			0.057
White	48 (48.5)	25 (37.8)	
African American	16 (16.6)	8 (12.1)	
Hispanic	13 (13.1)	21 (31.8)	
Asian	22 (22.2)	12 (18.1)	
Axial length, mm, mean ± SD	24.7 ± 1.5	23.9 ± 1.1	0.001
Central corneal thickness (μm, mean ± SD)	541.1 ± 41.2	554.7 ± 36.1	0.029
Intraocular pressure, mm Hg, mean ± SD	13.1 ± 3.8	14.1 ± 2.7	0.108
Retinal nerve fiber layer thickness (μm, mean±SD)	63.0 ± 14.8	95.6 ± 12.4	<0.001
VF mean deviation, dB, mean ± SD	−7.1 ± 5.2	−0.2 ± 1.2	<0.001
VF pattern standard deviation, dB, mean ± SD	7.9 ± 4.5	1.0 ± 0.1	<0.001

VF, visual field.

**Figure 3:**
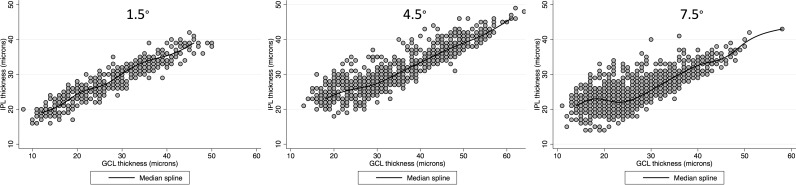
Bivariate scatter plots with the corresponding spline fits displaying the relationship of the IPL against ganglion cell layer thickness in 24 central, macular superpixels in glaucoma eyes as a function of eccentricity. Circle 1: 1.5° eccentricity; circle 2: 4.5° eccentricity; 7.5° eccentricity.

Figure 3 demonstrates scatter plots for the IPL versus GCL thickness according to eccentricity in glaucoma eyes with a spline fit. Below a GCL thickness cutoff point of approximately 30 μm, the IPL thickness tended to plateau and become relatively thicker than GCL thickness in superpixels located at 4.5° and 7.5° eccentricities (circles 2 and 3). Therefore, subsequent multivariable analyses predicting the IPL thickness were performed after excluding superpixels with a GCL thickness greater than 30 μm.

### Predictors of IPL Thickness in Healthy Subjects

We explored factors influencing the IPL thickness in healthy subjects. On univariable analyses, after accounting for correlation of superpixels within eyes, location at 4.5° eccentricity (β = 5.56, *P* < 0.001) was associated with thicker IPL in healthy subjects, whereas older age (β = –0.065; *P* = 0.011) and Asian ethnicity (β = –2.08; *P* = 0.042) predicted thinner IPL. Specifically, neither axial length (*P* = 0.419), sex (*P* = 0.312), or intraocular pressure (IOP; *P* = 0.185) demonstrated any correlation with IPL thickness. Asian eyes had significantly longer axial length (25.0 ± 1.4 mm) compared with white (24.4 ± 1.4 mm), African American (24.3 mm ± 1.4), and Hispanic patients (23.8 ± 1.2 mm). Hispanic (*P* = 0.03) and Asian (*P* = 0.034) eyes were significantly different from eyes belonging to white patients, whereas African American eyes were not (*P* = 0.888). Despite the fact that axial length was not correlated with the IPL thickness on univariable and multivariable analyses, it was included in the final multivariable analyses to account for its potential influence. On multivariable analyses with or without GCL in the model, older age (β = –0.080 to –0.099, *P* ≤ 0.002) and Asian ethnicity (β = –2.31 to –2.64, *P* = 0.012–0.002) were associated with thinner IPL, whereas being located at 4.5° eccentricity (β =3.03–5.51 with or without GCL thickness in the model, *P* < 0.001) was a predictor for thicker IPL. On the other hand, 7.5° eccentricity predicted lower IPL thickness (β = –3.04 and –3.01; *P* < 0.001 for both).

### Predictors of IPL Thickness in All Eyes

**Figure 4:**
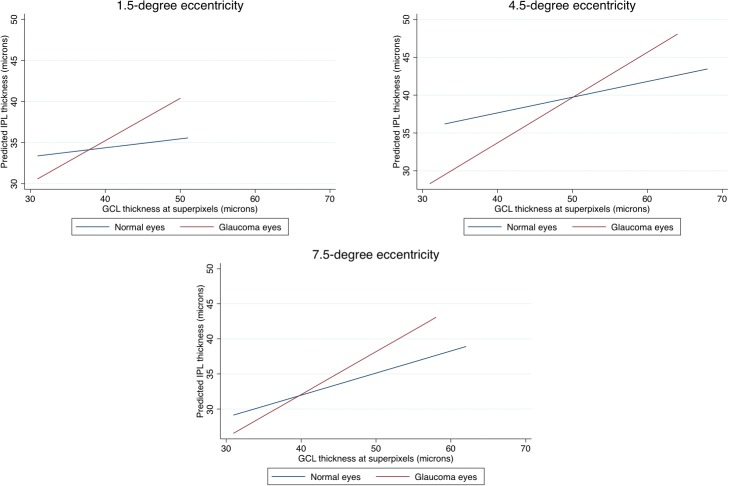
Graphs demonstrate the expected IPL thickness, as predicted by the multivariable model on the Y-axis as a function of GCL thickness plotted on the X-axis for macular superpixels located at 1.5° (top left), 4.5° (top right), and 7.5° (bottom) from the fovea. Superpixels with GCL thickness less than 30 μm were excluded as the linear relationship between the IPL and GCL thickness changed after this cutoff point.

**Figure 5:**
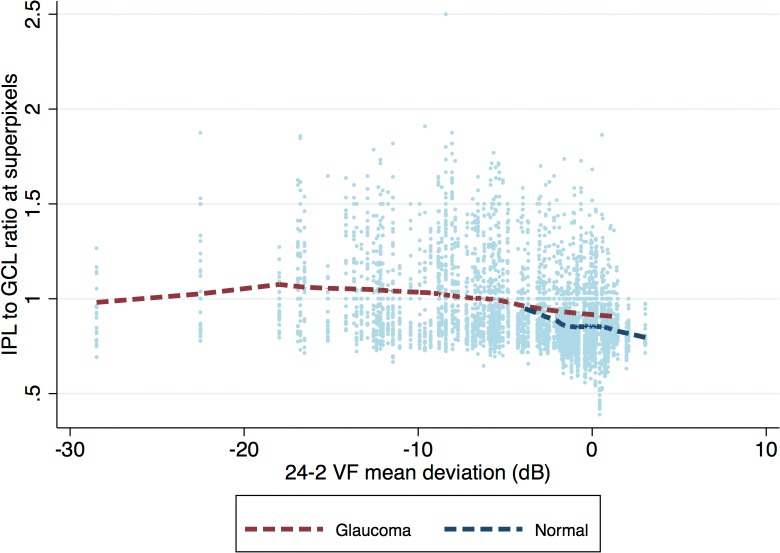
Scatter plot of IPL thickness to ganglion cell layer thickness ratio as a function of the MD on the 24-2 visual field with Lowess fit applied to healthy and glaucoma eyes separately.

Given the significant and variable effect of the eccentricity on the IPL thickness, we carried out separate multivariable analyses for prediction of IPL thickness for each eccentricity with all eyes included to investigate the effect of a diagnosis of glaucoma (Tables 2 and 3) on the IPL thickness. With multivariable mixed-model regression analyses, a diagnosis of glaucoma was associated with a different relationship between IPL and GCL thickness at all eccentricities after accounting for race and axial length. In other words, the interaction of GCL with diagnosis was statistically significant. Figure 4A through 4C shows the expected IPL thickness (on the y-axis) as predicted by the model against GCL thickness (plotted on the x-axis) for each level of eccentricity in glaucoma and healthy eyes. The slope of IPL thickness against GCL thickness was significantly steeper in glaucoma eyes compared with healthy eyes at all eccentricities. While glaucoma eyes tended to have a thinner IPL thickness, on average, for the same level of GCL thickness at the lower range of GCL thickness, this relationship reversed at higher GCL thickness levels (Fig. 4). When the analyses were repeated only on eyes with MD better than −6 dB on the 24-2 visual field, the results were very similar (data not shown). MD was not found to be a significant predictor when entered into multivariable analyses. We also explored the IPL to GCL thickness ratio as a function of diagnosis (Fig. 5). It can be observed that the Lowess fit for healthy eyes was consistently lower than that for glaucoma eyes, confirming that the IPL was actually relatively thicker in glaucoma eyes compared with healthy eyes at comparable ranges of GCL thickness. The results were the same when analyses were performed separately for different eccentricities. A multivariable logistic regression for identifying factors predictive of excluded superpixels showed that a diagnosis of glaucoma or having worse baseline MD (*P* < 0.001 for both), being located at 1.5° eccentricity (*P* < 0.001 compared with 4.5° eccentricity) or 7.5° eccentricity (*P* < 0.001 compared with 4.5° eccentricity and *P* = 0.055 compared with 1.5° eccentricity with 7.5° eccentricity having a higher exclusion rate) were predictors of having excluded superpixels.

**Table 2:** IPL and GCL Thickness Measurement for All Study Eyes and After Excluding Superpixels Where the GCL Thickness was <30 μm According to Eccentricity

	All Superpixels			Superpixels With GCL thickness >30 μm		
	Healthy	Glaucoma	P*	Healthy	Glaucoma	P*
IPL						
All points	34.4 ± 5.9	27.6 ± 6.1	<0.001	35.0 ± 5.7	33.2 ± 5.0	0.406
1.5° eccentricity, μm	34.1 ± 3.4	27.8 ± 5.9	<0.001	34.1 ± 3.4	34.1 ± 3.0	0.686
4.5° eccentricity, μm	39.6 ± 4.2	31.2 ± 6.5	<0.001	39.6 ± 4.2	35.2 ± 5.1	<0.001
7.5° eccentricity, μm	31.1 ± 4.9	25.2 ± 4.6	<0.001	31.6 ± 4.8	30.1 ± 3.6	0.465
GCL						
All points	41.3 ± 8.4	29.5 ± 10.1	<0.001	42.8 ± 7.5	39.8 ± 6.7	0.016
1.5° eccentricity, μm	36.9 ± 4.3	26.9 ± 9.7	<0.001	38.2 ± 4.7	37.8 ± 4.6	0.380
4.5° eccentricity, μm	49.7 ± 5.6	34.6 ± 11.4	0.013	49.8 ± 5.4	42.5 ± 7.4	<0.001
7.5° eccentricity, μm	37.2 ± 6.3	26.8 ± 7.9	<0.001	38.9 ± 5.4	36.8 ± 4.6	0.492

The latter is presented to be consistent with multivariable analyses predicting IPL thickness in all eyes; 1445 of 3960 (36.4%) superpixels had GCL thickness <30 μm.

* *P* values are based on multivariable linear mixed regression analyses entering variables with *P* < 0.15 in the model and accounting for the correlation of superpixels in each eye.

**Table 3:** Results of Multivariable Regression Analysis for Prediction of IPL Thickness in 99 Glaucoma Eyes and 66 Healthy Eyes at Superpixels Level as a Function of Eccentricity From the Fovea

	1.5° Eccentricity (Circle 1)		4.5° Eccentricity (Circle 2)	
	Coefficient (95%CI)	*P* Value	Coefficient (95%CI)	*P* Value
Age	–0.261 (−0.059, 0.006)	0.121	−0.035 (−0.071, 0.001)	0.059
Race (ref: non-Asian)	–0.849 (−1.583, −0.114)	0.024	−0.792 (−1.884, 0.299)	0.154
Axial length, mm	0.016 (−0.199, 0.231)	0.883	−0.049 (–0.312, 0.212)	0.708
GCL thickness, μm	0.900 (−0.010, 0.190)	0.077	0.161 (0.009, 0.314)	0.038
Glaucoma diagnosis	–18.033 (−21.853, −14.215)	<0.001	–17.623 (−25.030, −10.216)	<0.001
Glaucoma diagnosis * GCL thickness	0.483 (0.381, 0.586)	<0.001	0.352 (0.197, 0.507)	<0.001

**Table 3:** Extended

	7.5° Eccentricity (Circle 3)	
	Coefficient (95%CI)	*P* Value
Age	−0.046 (−0.806, −0.013)	0.007
Race (ref: non-Asian)	−0.080 (−1.002, 0.842)	0.864
Axial length, mm	−0.146 (−0.388, 0.094)	0.232
GCL thickness, μm	0.282 (0.177, 0.386)	<0.001
Glaucoma diagnosis	−6.263 (−10.043, −2.483)	0.001
Glaucoma diagnosis * GCL thickness	0.153 (0.042, 0.263)	0.007

We investigated the diagnostic performance of the IPL versus GCL thickness for discrimination of glaucomatous from healthy eyes. Table 4 demonstrates that for either global or superior/inferior macular hemiretinal regions, GCL thickness had higher AUCs (*P* = 0.056–0.277).

**Table 4:** AUCs (95%CI) for GCL and IPL Thickness Parameters for Discrimination of Glaucoma From Healthy Eyes

	GCL	IPL	*P* Value
Central macula (24 superpixels)	0.945 (0.912, 0.977)	0.919 (0.879, 0.958)	0.056
Superior hemiretina	0.942 (0.908, 0.975)	0.927 (0.890, 0.964)	0.277
Inferior hemiretina	0.885 (0.835, 0.935)	0.860 (0.805, 0.914)	0.148

All superpixels were included regardless of their GCL thickness for calculating the average central GCL thickness, superior hemiretina, and inferior hemiretina.

## Discussion

The goal of the current study was to investigate predictors of the IPL thickness in healthy eyes and to test the hypothesis that preferential IPL thinning, in comparison to GCL, could be detected with SD-OCT in glaucoma. We found that older age and Asian ethnicity were associated with thinner IPL. IPL thickness was higher at 4.5° eccentricity compared with the region located 1.5° from the fovea whereas it was thinner farther from the fovea (7.5° eccentricity). The IPL to GCL relationship varied as a function of diagnosis and the slope of IPL was significantly steeper in glaucomatous eyes at all eccentricities compared with healthy eyes. Results were consistent when analyses were limited to eyes with early glaucoma (MD > –6 dB). As confirmed by findings on Figure 5, there was no evidence of preferential IPL thinning in early stages of glaucoma; in fact, the Lowess fit for IPL was lower in normal eyes compared with glaucoma eyes. This is consistent with the lesser ability of the IPL thickness in discriminating glaucoma from healthy eyes compared with GCL thickness.

The utility of inner macular layer measurements for detection of early glaucoma is now well established. Macular OCT imaging provides a valuable tool for measuring various components of the RGC axonal complex in the central retina with excellent reproducibility.^4^,^13^,^16^ Previous studies have shown that macular RNFL, GCL, IPL, ganglion cell/inner plexiform layer (GCIPL), ganglion cell complex (GCC), and full retinal thickness measurements become thinner in glaucoma and can be used to detect evidence of glaucoma damage.^2^,^12^ An understanding of patterns of damage at the level of individual retinal layers in glaucoma could potentially benefit ongoing studies conducted to develop neuroprotective treatment strategies. Prior OCT studies focused on GCC or GCIPL measurements rather than individual inner retinal layer measurements due to issues with image quality and lack of reliable segmentation algorithms. A few reports to date have studied GCL and IPL thickness as isolated layers. Tan et al.^8^ and Moura and colleagues^14^ showed that IPL and GCL thinning could be measured in glaucomatous eyes.

The effect of chronic elevation of IOP on the morphology of single ganglion cells has been described in animals and the negative influence of increased IOP on RNFL thickness and concomitant RGC loss has been demonstrated.^11^,^17^,^18^ These studies showed that GCL thickness was a good surrogate for RGC density in the macula.^11^,^17^,^18^ GCL measurements have been found to be able to discriminate glaucoma from healthy eyes in clinical setting and to be superior to IPL thickness in this regard.^12^,^13^ There is increasing evidence that morphologic alterations of RGC dendrites occur before changes in the RGC somas or axons.^11^,^17^,^19^ As a response to injury, RGCs begin to pare their dendrites to preserve energy at the level of the cell soma. Over time, many of the damaged axons undergo retrograde degeneration and finally, the soma itself begins to shrink.^10^,^11^ Our data do not support the hypothesis that the early dendritic shrinkage or pruning is detectable with current OCT technology. This finding would be most evident in early glaucoma; however, as seen on the modeled relationship of the IPL versus GCL thickness on Figure 4, the IPL thickness actually tended to be relatively higher in glaucoma eyes compared with healthy eyes at superpixels demonstrating higher GCL thickness measurements (i.e., superpixels with healthier RGCs), a finding that does not support our hypothesis. Figure 5 also provides a confirmation for this finding. One possible explanation for our findings is that given the slow course of glaucoma, only a very small proportion of RGCs are undergoing dendritic shrinkage in anticipation of apoptotic changes in the RGC somas and therefore, this small proportion of RGCs scattered across the macula and the rest of the retina would not be detectable given the limitations of current OCT technology.

We found that Asian ethnicity predicted a thinner IPL, whereas being located at 4.5° eccentricity was a predictor for thicker IPL in healthy subjects, and hence, those predictors were entered as confounding variables in subsequent multivariable analyses in addition to other potential clinical factors influencing the IPL thickness in the entire group. The latter analyses demonstrated that that the relationship between IPL and GCL (i.e., the IPL/GCL slope) was consistently different in glaucoma versus healthy eyes at all eccentricities. In the more severe stages of the disease (GCL thickness ∼≤30 μm), IPL reached a measurement floor and the linear relationship of IPL and GCL flattened in glaucoma eyes at 4.5° and 7.5° eccentricities.

Distance from the foveal center was found to be an important factor influencing the IPL to GCL relationship, and hence, the multivariable analyses were separately repeated for each eccentricity; however, the results were consistent regardless of eccentricity. This is despite the fact that the individual inner retinal layers are very thin around the foveal center and therefore, measurements are more prone to noise compared with more peripheral superpixels. A limitation of our approach is that the size of superpixels used (3° × 3°) was relatively large compared with the steep changes in GCL and IPL layer thickness in the perifoveal area leading to significant averaging of thickness values. In this area, GCL is proportionally thicker than IPL in healthy eyes.^14^,^20^ It has been shown that from approximately 3° outward, the IPL thickness changes are less dramatic than GCL thickness, which reaches a peak around 4.5° from the fovea. Previous studies have confirmed that the GCL and IPL displayed different topographic patterns.^21^,^22^

In multivariable models predicting IPL thickness, thinner GCL, Asian ethnicity, and older age were associated with thinner IPL. One important finding was that the magnitude of the influence of these factors was small (2.3–2.6 μm for Asian ethnicity, ∼1 μm for each decade, and 3–5 μm for eccentricity), whereas GCL thickness was associated much more strongly with the IPL thickness. Previous studies exploring predictive factors for GCIPL thickness reached similar conclusions.^6^,^23^ In a study by Koh et al.,^23^ older age, female sex, longer axial length, and thinner RNFL thickness were associated with thinner GCIPL layer. However, in another study, the GCIPL layer was thinner in men.^24^ Mwanza et al.^6^ reported that GCIPL was thinner in subjects of European descent and in men. In line with studies by Curcio et al.^25^ and Moura and colleagues,^14^ IPL thickness was positively associated with 4.5° eccentricity and tended to be thinner at 7.5° eccentricity regardless of diagnosis.

The significantly steeper relationship of IPL versus GCL in glaucoma eyes likely reflects the concomitant and related loss of these layers in glaucoma leading to a wider range of measurements and higher correlation in glaucoma eyes. The IPL to GCL slope in glaucoma eyes was steepest at 4.5° eccentricity, which is probably a result of the higher relative IPL thickness in this region.

IPL thickness has not been found to outperform GCL for diagnosing glaucoma.^12^,^13^,^26^ Several factors may contribute to this finding, including higher interindividual variability and the smaller dynamic range for IPL thickness. The composition of IPL may also explain lower performance of IPL in detecting early glaucoma.^12^ The IPL consists not only of RGC dendrites but also of cell processes of amacrine and bipolar cells.^27^ As glaucoma is not expected to be associated with bipolar cell or glycinergic amacrine cells loss,^28^ lack of changes in these cells may lessen the apparent impact of IPL thinning, potentially increasing the measurement floor for IPL.^13^ We also found that GCL thickness (global, superior, or inferior hemiretinal regions) performed better than IPL thickness for discriminating glaucoma from healthy eyes. This finding is consistent with the fact that preferential IPL thinning could not be proven in glaucoma eyes. Nevertheless, the discrimination ability of IPL was excellent and comparable to that of GCL. It is possible that different regions of the macula may undergo glaucomatous damage in different ways at various points early during the disease and therefore, IPL thickness may provide additional information to that gleaned from GCL thickness. A recent study showed that each macular layer has a characteristic region with the best glaucoma diagnostic capability and that segmentation of individual macular layers may enhance diagnosis of glaucoma along the spectrum of severity glaucoma.^12^

The main focus of the current study was the respective changes in local IPL and GCL thickness measurements in early to moderate stages of glaucoma. The IPL thickness reached its measurement floor with advancing damage sooner than GCL (about the time when GCL thickness reached 30 μm); therefore, IPL thickness seems to be less relevant clinically in eyes with advanced glaucoma. This is consistent with prior findings reported by Miraftabi et al.^2^ Superpixels belonging to eyes with glaucoma, those with worse MD, and those located at 1.5° or 7.5° eccentricity were more likely to be excluded from the analysis. However, this is unlikely to have affected our main results as superpixels with early evidence of damage (i.e., those located to the right of the graph on Fig. 5) were the least likely superpixels to have been excluded.

There were several limitations to this study. The study sample included eyes with a range of glaucoma severity. However, when only eyes with early glaucoma (MD > –6 dB on 24-2 visual fields) were included, the results were very consistent (data not shown). Confounding noise inherent in segmentation of the individual layers of the retina, including IPL, is also a potential limitation of the current study that will likely be addressed in the near future as the quality of OCT imaging improves. Age and ethnic distribution of the normal and glaucoma groups was somewhat different in this study; although this was accounted for in multivariable analyses, investigating this topic in a group of healthy and glaucoma eyes matched by age and ethnicity would be of interest. Inclusion of some diabetic patients without retinopathy may have introduced a confounding effect on the results; there is some evidence that mild inner retinal thinning can be present before overt signs of diabetic retinopathy occur.^12^

Our study was based on cross-sectional data and therefore, does not definitively rule out preferential thinning of the IPL. The IPL to GCL relationship needs to be studied longitudinally to better understand how the corresponding layer thickness measurements evolve over the course of glaucoma. We used 3° × 3° superpixels in preference to the Early Treatment Diabetic Retinopathy Study (EDTRS) grid as the latter was originally designed for retinal diseases (specifically diabetic retinopathy) and is not considered well suited for studying glaucoma as its defined sectors cross the temporal raphe and therefore, anatomically do not correspond well to the expected pattern of glaucoma damage. Also, the ETDRS sectors are fairly large, and the resulting averaging of data can mask findings that may be observed when local structural relationships are evaluated in more detail within smaller areas (such as 3° × 3° superpixels). Other OCT devices provide macular thickness measurements within wedge-shaped sectors around the fovea. We were not able to carry out similar measurements as the data are provided in a different format by Spectralis SD-OCT.

In conclusion, we did not find any evidence of preferential IPL thinning compared with GCL in glaucoma eyes in the macular region. This finding needs to be confirmed in eyes with preperimetric glaucoma and in longitudinal studies.
